# *GATA3* somatic mutations are associated with clinicopathological features and expression profile in TCGA breast cancer patients

**DOI:** 10.1038/s41598-020-80680-9

**Published:** 2021-01-18

**Authors:** Fahimeh Afzaljavan, Ayeh Sadat Sadr, Sevtap Savas, Alireza Pasdar

**Affiliations:** 1 Department of Medical Genetics and Molecular Medicine, Faculty of Medicine, Mashhad University of Medical Sciences, Mashhad, Iran; 2 Aquaculture Research Center- South of Iran, Iranian Fisheries Science Research Institute, Agricultural Research Education and Extension Organization (AREEO), Ahvaz, Iran; 3 Discipline of Genetics, Faculty of Medicine, Memorial University, St. John’s, NL Canada; 4 Discipline of Oncology, Faculty of Medicine, Memorial University, St. John’s, NL Canada; 5 Division of Applied Medicine, Medical School, University of Aberdeen, Foresterhill, Aberdeen, AB25 2ZD UK; 6 Bioinformatics Research Group, Mashhad University of Medical Sciences, Mashhad, Iran

**Keywords:** Cancer, Computational biology and bioinformatics, Genetics, Molecular biology, Biomarkers, Oncology, Risk factors

## Abstract

The effect of somatic mutations and the gene expression profiles on the prognosis is well documented in cancer research. This study was conducted to evaluate the association of *GATA3* somatic mutations with tumor features, survival, and expression profiles in breast cancer. Clinicopathological information was compared between TCGA-BRCA patients with *GATA3*-mutant and non-mutant tumors in all patients as well as in ER-positive subgroup. Cox-regression method was used to evaluate the association of the *GATA3* mutation status with overall survival time. Differential gene expression, functional annotation, and protein–protein interaction analyses were performed using edgeR, Metascape, DAVID, STRING and CytoNCA. *GATA3*-mutant and non-mutant samples had significantly different clinicopathological features (*p* < 0.05). While *GATA3* mutation status was not associated with the overall survival in the entire cohort (*p*_*adj*_ = 0.52), the *GATA3-*wild type ER-positive cases had a better prognosis than mutant ones (*p*_*adj*_ = 0.04). *GATA3* expression was higher in tumors than normal tissues. Several pathways were different between mutant and non-mutant groups (*p* < 0.05). Interleukin-6 was found as the highest scored gene in both comparisons (normal vs. mutant and normal vs. non-mutant groups) in the entire patient and in the ER-positive subgroup, suggesting the association of IL6 with breast tumorigenesis. These findings suggest that *GATA3* mutations can be associated with several tumor characteristics and influence the pattern of gene expression. However, *GATA3* mutation status seems to be a prognostic factor for the disease only in ER-positive patients.

## Introduction

Breast cancer, the most common type of cancer in women worldwide, is a heterogeneous disease with different pathological and molecular features and subtypes^1^. The disease is caused by both environmental and genetic factors^2^. In this regard, numerous genetic risk factors have been identified for tumor development and progression^3^. Except for the genes with highly penetrant and hereditary mutations, such as *BRCA1* and *BRCA2*^4^, the genetic basis of breast cancer and the role of genetic variations and their effects on malignant transformation are currently complex and requires further investigations. Several studies have demonstrated that somatic mutations in oncogenes and tumor suppressor genes are major drivers of different types of breast tumors and correlate with clinicopathological characteristics of the disease, response to therapy, or prognosis^5–7^. GATA binding protein 3 (*GATA3*) is one of the important genes involved in breast cancer development^8^.

GATA binding protein 3 is a transcription factor that encodes a protein member of the GATA family. GATA family members have two conserved Zinc-finger DNA binding domains. This transcription factor binds to promoters of target genes through the consensus (A/T)GATA(A/G) motifs^9^. Previous studies have demonstrated that GATA3 protein has crucial roles in cell development and differentiation in different types of cells, including mammary tissue^10^. Therefore, variations in its expression can affect downstream pathways and result in changes in cellular characteristics as its higher expression has been identified in hormone receptor-positive breast cancer patients^11^. While some data have pointed out that the *GATA3* expression level is not an independent prognostic factor^11^, several researchers have reported that it was associated with better survival in breast cancer patients^12,13^. Also, it has been reported that breast tumors expressing low levels of *GATA3* were correlated with larger tumors^14^. A literature report suggests that related pathways may be the reason for the association of this gene with some clinical features of breast cancer^15,16^. In light of these findings, *GATA3* has been considered as an important gene in breast development and cancer^17^. However, the role of *GATA3* somatic mutations in the development of breast tumor characteristics, patient survival outcomes, and its impact on tumor gene expression profiles is poorly understood.

In this study, we evaluated the genomic alterations of *GATA3* in breast tumors, using the data collected by TCGA^18^, and analyzed the associations of *GATA3* somatic mutations with tumor features, patient survival, and tumor gene expression profiles to highlight the clinical importance of this gene in breast cancer.

## Results

### *GATA3* somatic mutation status and association with clinicopathological features

In the TCGA-BRCA cohort, tumors of 975/1085 female patients were evaluated for somatic mutations. Among these patients, a total of 103 different *GATA3* mutations were identified in 138 patients (14.15%). Insertions constituted the largest type of mutations (50.5%), followed by deletions (29.1%) and substitutions (20.4%). A large portion of the mutations (74.7%) resulted in frame-shifts and variant effect predictor (VEP)^19^ has indicated 96.3% of all mutations were predicted to have a high or moderate impact. The most frequent mutation was X309, which is a two-base pairs (CA) deletion/splice site mutation (chr10:g.8069470delCA, annotated as *GATA3* X309_splice in the GDC portal). This mutation was detected in tumors from 21 patients (15.22% of patients with *GATA3* mutations). There were 11 additional recurrent mutations identified in more than one patient (n = 2–8), while the rest of the mutations were detected only in one patient.

The average diagnosis age was 45.66 ± 13.65 and 58.77 ± 12.97 in patients with and without *GATA3* mutations, respectively (*p* = 0.001; Table 1). We compared the *GATA3* mutation status in patients with different age categories. This analysis showed that the proportion of the patients with *GATA3* mutated tumors was higher in the patients diagnosed under 40 years of age compared to those who were diagnosed after 40 years of age [20 of 89 patients under 40 years old (22.5%) and 118 of 885 patients above 40 years old (13.3%), respectively; *p* = 0.02]. In addition to age at diagnosis, menopausal status was significantly different between patients with and without *GATA3* mutations (*p* = 0.00004; Table 1). Other clinicopathological characteristics that were associated with *GATA3* mutation status in this patient cohort (Table 1) are the following: pathologic tumor size was significantly different between patients with *GATA3* mutant tumors compared to patients with wild-type *GATA3* tumors (*p* = 0.01). A significant difference was also seen with tumor histological types. There was a strong relationship between the *GATA3* mutation and ER/PR status; almost none of the tumors with *GATA3* mutations were ER-negative (Table 1). Additionally, in the multivariable logistic regression analysis, age at diagnosis, tumor size (pT), PR status, and histological tumor type were found to be independently associated factors of *GATA3* mutation status in breast cancer (Table 2).

**Table 1:** Results of univariate logistic regression analysis examining the association between *GATA3* mutation status and clinical features.

Variables	All patients				ER-Positive patients			
Categories	Wild n (%)	Mutant n (%)	*p* value^a^	OR (95% CI)	Wild n (%)	Mutant n (%)	*p* value^a^	OR (95% CI)
**Age**								
Mean	58.77 ± 12.97	45.66 ± 13.65	**0.001**	0.98 (0.96–0.99)	60.03 ± 13.02	54.81 ± 13.68	**0.00005**	0.09 (0.95–0.98)
Age ≤ 35	25 (3%)	6 (4.3%)			16 (2.8)	5 (3.9)		
Age > 35	811 (97%)	14 (95.7%)	0.40	0.68 (0.27–1.68)	565 (97.2)	123 (96.1)	0.489	1.43 (0.52–3.99)
Age ≤ 40	69 (8.3%)	20 (14.5%)			39 (6.7)	19 (14.8)		
Age > 40	767 (91.7%)	118 (85.5%)	**0.02**	0.53 (0.31–0.91)	542 (93.3)	109 (85.2)	**0.003**	2.42 (1.35–4.35)
**Menopause status**								
Peri and Pre	193 (25.6%)	53 (43.8%)			123 (23.3)	49 (43.4%)		
Post	562 (74.4%)	68 (56.2%)	**0.00004**	0.44 (0.30–0.65)	405 (76.7)	64 (56.6)	**0.00002**	2.52 (1.61–3.85)
**Race**								
White	577 (75.8%)	95 (73.1%)			418 (81.2)	85 (70.8)		
Black/African-American	138 (18.1%)	22 (16.9%)	0.90	0.97 (0.59–1.60)	75 (14.5)	22 (18.3)	0.175	1.44 (0.85–2.45)
Asian	46 (6.1%)	13 (10%)	0.10	1.72 (0.89–3.30)	22 (4.3)	13 (10.8)	**0.004**	2.91 (1.41–5.99)
**History of other malignancy**								
No	783 (93.7%)	132 (95.7%)			537 (92.4)	122 (95.3)		
Yes	53 (6.3%)	6 (4.3%)	0.37	0.67 (0.28–1.59)	44 (7.6)	6 (4.7)	0.253	0.60 (0.25–1.44)
**History of neoadjuvant therapy**								
No	826 (98.8%)	135 (98.5%)			572 (98.3)	125 (98.4)		
Yes	10 (1.2%)	2 (1.5%)	0.80	1.22 (0.27–5.65)	10 (1.7)	2 (1.6)	0.910	0.91 (0.20–4.23)
**Margin status**								
Negative	698 (89.9%)	117 (88%)			482 (89.1)	108 (87.1)		
Positive/Close	78 (10.1%)	16 (12%)	0.49	1.22 (0.69–2.17)	59 (10.9)	16 (12.9)	0.526	1.21 (0.67–2.18)
**Number of involved lymph node**								
Median (Q1–Q3)	2.28 ± 4.46	1.95 ± 3.31	0.45	0.98 (0.93–1.03)	1 (0–3)	1 (0–3)	0.284	0.97 (0.91–1.03)
**Lymph node ratio**								
Median (Q1–Q3)	0.16 ± 0.26	0.18 ± 0.27	0.56	1.25 (0.61–2.57)	0.06 (0.0–0.25)	0.06 (0.0–0.24)	0.756	0.88 (0.39–1.98)
**Lymph node ratio category**								
Negative = 0	349 (49.4%)	53 (46.5%)			218 (44.8)	48 (45.7)		
Low (> 0–0.2)	179 (25.3%)	29 (25.4%)	0.79	1.07 (0.66–1.74)	136 (27.9)	29 (27.6)	0.902	0.97 (0.58–1.61)
Intermediate (> 0.2–0.65)	118 (16.7%)	23 (20.2%)	0.36	1.28 (0.75–2.19)	89 (18.3)	21 (20.0)	0.812	1.07 (0.61–1.79)
High (> 0.65)	61 (8.6%)	9 (7.9%)	0.94	0.97 (0.46–2.07)	11 (9.0)	7 (6.7)	0.457	0.71 (0.31–1.70)
**AJCC pT**								
T1 and T2	717 (85.9%)	106 (77.4%)			496 (85.4)	99 (78.0)		
T3 and T4	118 (14.1%)	31 (22.6%)	**0.01**	1.78 (1.14–2.77)	85 (14.6)	28 (22.0)	**0.040**	1.65 (1.02–2.66)
**AJCC pN**								
Negative	398 (48.4%)	62 (46.6%)			256 (44.8)	57 (46.0)		
Positive	424 (51.6%)	71 (53.4%)	0.70	1.07 (0.74–1.55)	315 (55.2)	67 (54.0)	0.818	0.95 (0.65–1.41)
**AJCC pM**								
Negative	702 (97.2%)	115 (97.5%)			492 (98.6)	105 (97.2)		
Positive	15 (2.1%)	3 (2.5%)	0.76	1.22 (0.35–4.28)	7 (1.4)	3 (2.8)	0.318	2.01 (0.51–7.89)
**AJCC stage**								
Stage 1 and 2	627 (76.7%)	96 (71.1%)			428 (75.1)	90 (72.0)		
Stage 3 and 4	191 (23.3%)	39 (28.9)	0.16	1.33 (0.89–2.00)	142 (24.9)	35 (28.0)	0.473	1.17 (0.76–1.81)
**ER status by IHC**								
Negative	216 (27.1%)	1 (0.8%)			–	–	–	–
Positive	582 (72.9%)	128 (99.2%)	**0.0001**	47.51 (6.60–341.94)	–	–	–	–
**PR status by IHC**								
Negative	282 (35.5%)	24 (18.5%)			83 (14.3)	23 (18.0)		
Positive	513 (64.5%)	106 (81.5%)	**0.0002**	2.43 (1.52–3.87)	497 (85.7)	105 (82.0)	0.295	0.76 (0.46–1.27)
**HER2 status**^**b**^								
Negative	589 (81.5%)	91 (85%)			431 (81.6)	91 (85.0)		
Positive	134 (18.5%)	16 (15%)	0.37	0.77 (0.44–1.36)	97 (18.4)	16 (15.0)	0.400	0.78 (0.4401.39)
**Receptor status**^**c**^								
ER and/or PR positive	598 (77.3%)	129 (100%)			–	–	–	–
HER2 overexpressed	32 (4.1%)	0 (0%)	1.00	0.00 (–)	–	–	–	–
TNBC	144 (18.6%)	0 (0%)	1.00	0.00 (–)	–	–	–	–
**Anatomic neoplasm subdivision**								
Left	432 (51.6%)	75 (54.3%)			299 (51.4)	69 (53.9)		
Right	405 (48.4%)	63 (45.7%)	0.55	0.90 (0.62–1.29)	283 (48.6)	59 (46.1)	0.604	0.90 (0.62–1.33)
**Histological type of tumor**^**d**^								
IDC	617 (73.8%)	103 (74.6%)			400 (68.7)	96 (75.0)		
ILC	148 (17.7%)	14 (10.1%)	0.06	0.57 (0.32–1.02)	136 (23.4)	14 (10.9)	**0.005**	0.43 (0.24–0.78)
Other	71 (8.5%)	21 (15.2%)	**0.03**	1.77 (1.04–3.01)	46 (7.9)	18 (14.1)	0.104	1.63 (0.91–2.94)

*AJCC* American Joint Committee on Cancer, *CI* confidence interval, *ER* estrogen receptor, *IDC* invasive ductal carcinoma, *IHC* immunohistochemistry, *ILC* invasive lobular carcinoma, *ISH* in situ hybridization, *OR* odds ratio, *PR* progesterone receptor, *TNBC* triple negative breast cancer.

^a^Significant p values are shown in bold.

^b^According to ISH/IHC results.

^c^Association between the receptor status and *GATA3* mutation status cannot be estimated because all *GATA3* mutant tumors are also ER and/or PR positive. Significant *p* values are shown in bold.

^d^Other category includes rare types of tumors (e.g. Metaplastic, Medullary tumors).

**Table 2:** Results of the multivariable logistic regression analysis.

All patients			ER-Positive patients		
Variable	*p* value	OR (95% CI)	Variable	*p* value	OR (95% CI)
Age at diagnosis	**0.00040**	0.97 (0.96–0.99)	Age at diagnosis	**0.001**	0.97 (0.96–0.99)
Tumor size (T3 and T4 vs. T1 and T2)	**0.00262**	2.09 (1.29–3.38)	Tumor size (T3 and T4 vs. T1 and T2)	0.195	1.45 (0.83–2.45)
Histological type^a^	**0.00513**		Histological type		
ILC versus IDC	**0.00968**	0.44 (0.23–0.82)	ILC versus IDC	**0.011**	0.42 (0.21–0.81)
Other type versus IDC	0.12139	1.59 (0.88–2.86)	Other type versus IDC	0.075	1.76 (0.94–3.30)
PR status (positive vs. negative)	**0.00001**	2.92 (1.81–4.73)	**Race**		
-	-	-	Black/African American versus White	0.285	1.35 (0.78–2.34)
-	**-**	-	Asian versus White	**0.031**	2.29 (1.08–4.86)

Significant *p* values are shown in bold.

*CI* confidence interval, *IDC* invasive ductal carcinoma, *ILC* invasive lobular carcinoma, *OR* odds ratio, *PR* progesterone receptor.

^a^Other category includes rare histological types, such as metaplastic and medullary tumors.

We repeated these analyses in the ER-positive subgroup (Tables 1, 2). Overall, the results in this subgroup analysis were similar to that of the entire patient cohort. An interesting finding in the ER-positive subgroup analysis was that the mutant cases were more frequently presented than non-mutants in the Asian population (Table 1).

### *GATA3* somatic mutations and prognosis

The median overall survival was 10.80 ± 0.7 years (11.69 ± 3.63 and 10.61 ± 2.19 years in patients without *GATA3* mutation compared with patients with *GATA3* mutation, respectively; *p* = 0.73). There was no significant difference between the two groups in terms of median survival time. This finding was also similar in the ER-positive subgroup (Table 3).

**Table 3:** Results of the univariate and multivariable Cox regression analysis for *GATA3* mutation status.

Cox regression	All patients			ER-Positive patients		
	Variable	*p* value	HR (95% CI)	Variable	*p* value	HR (95% CI)
Univariate	*GATA3* mutation status (yes vs. no)	0.73	1.09 (0.66–1.80)	*GATA3* mutation status (yes vs. no)	0.40	1.26 (0.74–2.15)
Multivariable	*GATA3* mutation status (yes vs. no)	0.52	1.22 (0.66–2.26)	*GATA3* mutation status (yes vs. no)	**0.040**	1.84 (1.03–3.27)
	Age at diagnosis	**0.0001**	1.03 (1.02–1.05)	Age at diagnosis	**2.411E−8**	1.05 (1.03–1.07)
	Stage category (S3 and S4 vs. S1 and S2)	**7.461E−10**	4.11 (2.62–6.44)	Stage category (S3 and S4 vs. S1 and S2)	**0.026**	1.90 (1.09–3.33)
	Radiation therapy status (yes vs. no)	**0.003**	0.49 (0.31–0.79)	Lymph node status category (positive vs. negative)	0.104	1.63 (0.90–2.96)

*CI* confidence interval, *HR* hazards ratio.

Univariate Cox proportional hazard analysis indicated age at diagnosis, menopause status, lymph node ratio, history of neoadjuvant therapy and adjuvant radiation therapy to be associated with survival times in the patients. Also, several tumor characteristics including margin status, pathologic tumor size (pT), lymph node (pN), and stage were associated with overall survival (Table S2).

While Multivariable Cox regression model adjusting for prognostic factors revealed that *GATA3* somatic mutation status was not an independent prognostic factor for all patients (*p*_*adj*_ = 0.52), wild type samples indicated better prognosis in the ER-positive subgroup (*p*_*adj*_ = 0.04) (Table 3). However, age (*p*_*adj*_ = 0.0001), stage (*p*_*adj*_ = 7.461E−10) and radiation therapy (*p* = 0.003) were significantly and independently associated with overall survival time in the entire patient cohort. Analysis of the ER-positive cases indicated age (*p*_*adj*_ = 2.411E−8) and stage (*p*_*adj*_ = 0.026) as independent factors associated with overall survival time.

### Gene expression analysis

According to the TCGA expression data, *GATA3* expression level was higher in *GATA3*-mutant (log FC = 2.78, *p* = 4.38E−34 in all patients and log FC = 2.66, *p* = 2.07E−57 in ER-positive subgroup) and non-mutant (log FC = 1.76, *p* = 2.11E−21 in all patients and log FC = 1.96, *p* = 3.24E−46 in ER-positive subgroup) tumors than normal tissues. While mutant tumors had a higher level than non-mutants (log FC = 1.02; *p* = 1.15E−12), this was not detected in the analysis of the ER-positive breast cancer patients.

**Figure 1:**
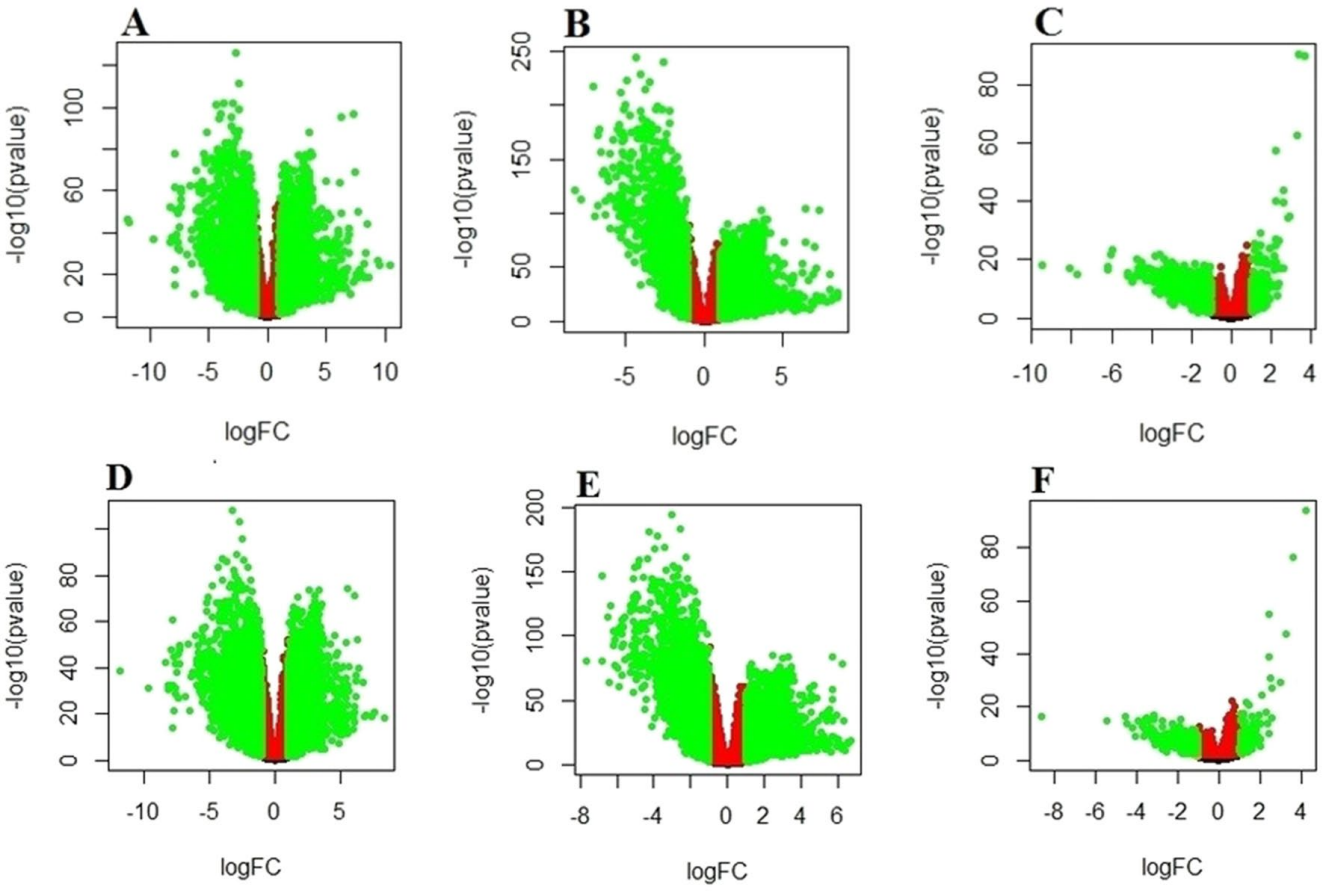
Volcano plats showed analysis of differential expressed genes (DEGs) between the normal compared with the tumors (*GATA3*-mutant and non-mutant). (**A**) Log_2_-fold change mutant and normal; (**B**) non-mutant and normal; (**C**) non-mutant and mutant; (**D**) Log_2_-fold change mutant and normal in ER-positive patients; (**E**) non-mutant and normal in ER-positive patients; (**F**) non-mutant and mutant in ER-positive patients. Green dots represent significantly DEGs (FDR < 0.05 and log FC > 1).

A total of 4816 differentially expressed genes (DEGs) were observed between the *GATA3*-mutant and normal tissues (2476 up-regulated and 2340 down-regulated genes). Additionally, there were a total of 4308 DEGs between the *GATA3*-non-mutant and normal tissues (2593 up-regulated and 1715 down-regulated genes). Finally, 907 DEGs between the non-mutant and mutant tumors were found: 169 genes were up-regulated and 738 genes were down-regulated at an FDR < 0.05 and log fold change (log FC) > 1. In the ER-positive subgroup, 4522 (2143 up-regulated and 2379 down-regulated genes), 4066 (2055 up-regulated and 2011 down-regulated genes) and 480 genes (103 up-regulated and 377 down-regulated genes) were found in the comparison between mutant versus normal, non-mutant versus normal and non-mutant versus mutant tumors, respectively. Volcano plots are shown in Fig. 1.

The most up and down-regulated DEGs in three categories of comparison are listed in Table 4. *MYH2* and *CKM* in mutant versus normal and non-mutant versus normal and *SMR3B* in mutant versus non-mutant were the top down-regulated genes. The top up-regulated genes were *MUC2*, *S100A7A* and *ALDOB* in mutant versus normal, non-mutant versus normal and mutant versus non-mutant, respectively. The ER-positive subgroup analysis showed *MUC2*, *CST5* and *ALDOB* as the top up-regulated genes and *MYH2* as the top down-regulated gene between mutant versus normal, non-mutant versus normal*,* and *CSN1S1* as the top down-regulated gene between mutants versus non-mutant samples.

**Table 4:** Differentially expressed genes (DEGs) between *GATA3*-mutant versus normal and *GATA3*-non-mutant versus normal and *GATA3*-mutant versus non-mutant tissues according to the TCGA data.

	Gene name	Fold change	FDR	Gene name	Fold change	FDR
*GATA3* mutant versus normal	All patients: 4816 (2476 up-regulated and 2340 down-regulated genes)			ER-Positive patients: 4522 (2143 up-regulated and 2379 down-regulated genes)		
Up-regulated DEGs	MUC2	10.43879	3.33E−24	MUC2	8.329625376	2.62 E−18
	CGA	9.492646	3.97 E−24	CHRNA9	7.628457572	7.85 E−21
	CHRNA9	9.389171	1.25E−26	CGA	7.332730083	1.31E−18
	CPLX2	8.522081	5.63E−19	PCSK1	6.922966106	1.79E−19
	CST4	8.506592	2.09E−43	TRH	6.85432771	8.50E−19
Down-regulated DEGs	MYH2	− 11.8785	1.83E−45	MYH2	− 11.8811779	6.71E−38
	CKM	− 11.8163	3.02E−44	NRAP	− 9.667105996	8.76E−31
	NRAP	− 9.63614	4.01E−36	TNNC2	− 8.329242401	9.99E−42
	TNNC2	− 8.32733	3.04E−51	TCAP	− 8.199816299	5.68E−32
	ACTA1	− 8.21929	4.09E−35	ACTA1	− 8.118831848	4.32E−30
*GATA3* non-mutant versus normal	All patients: 4308 (2593 up-regulated and 1715 down-regulated genes)			ER-Positive patients: 4066 (2055 up-regulated and 2011 down-regulated genes)		
Up-regulated DEGs	S100A7A	8.467232	9.82E−22	CST5	6.658392275	1.67E−18
	CSAG1	8.423582	3.50E−26	S100A7A	6.546948974	1.96E−16
	CST5	8.403968	2.03E−22	CGA	6.525469762	1.16E−17
	CGA	8.399469	2.10E−22	CARTPT	6.349247937	1.00E−10
	MAGEA12	8.173768	5.55E−22	CST4	6.32237147	7.11E−34
Down-regulated DEGs	MYH2	− 8.18882	1.49E−120	MYH2	− 7.688190533	6.83E−80
	CKM	− 7.80643	4.34E−112	PYGM	− 6.809240454	7.50E−145
	PYGM	− 7.07911	1.61E−215	ACTA1	− 6.782578399	4.19E−79
	NRAP	− 6.89436	1.09E−95	ATP2A1	− 6.539245722	2.40E−113
	ATP2A1	− 6.75453	8.90E−171	NRAP	− 6.423415223	1.04E−63
*GATA3* mutant versus non-mutant	All patients: 907 (169 up-regulated and 738 down-regulated genes)			ER-Positive patients: 480 (103 up-regulated and 377 down-regulated genes)		
Up-regulated DEGs	ALDOB	3.731806	9.25E−87	ALDOB	4.193275263	1.63E−90
	AMY2A	3.414509	9.25E−87	AMY2A	3.62447513	3.97E−73
	C8orf34	3.308972	2.16E−59	C8orf34	3.23103563	1.77E−44
	ZPLD1	2.907425	4.75E−32	ZPLD1	2.950533096	9.72E−27
	LOC284749	2.889682	1.42E−31			
Down-regulated DEGs	SMR3B	− 9.48864	3.11E−16	CSN1S1	− 8.610397883	1.76E−14
	CSN1S1	− 8.13224	3.62E−15	MYOC	− 5.400766258	8.60E−13
	CSN3	− 7.72831	1.09E−13	MSLN	− 4.537413853	3.42E−14
	C4orf7	− 6.20695	1.00E−14	DMBT1	− 4.476329021	4.97E−12
	FABP7	− 6.20416	7.96E−16	MYH2	− 4.192987371	2.92E−10
				SMR3B	− 4.029963607	1.15E−07

**Figure2:**
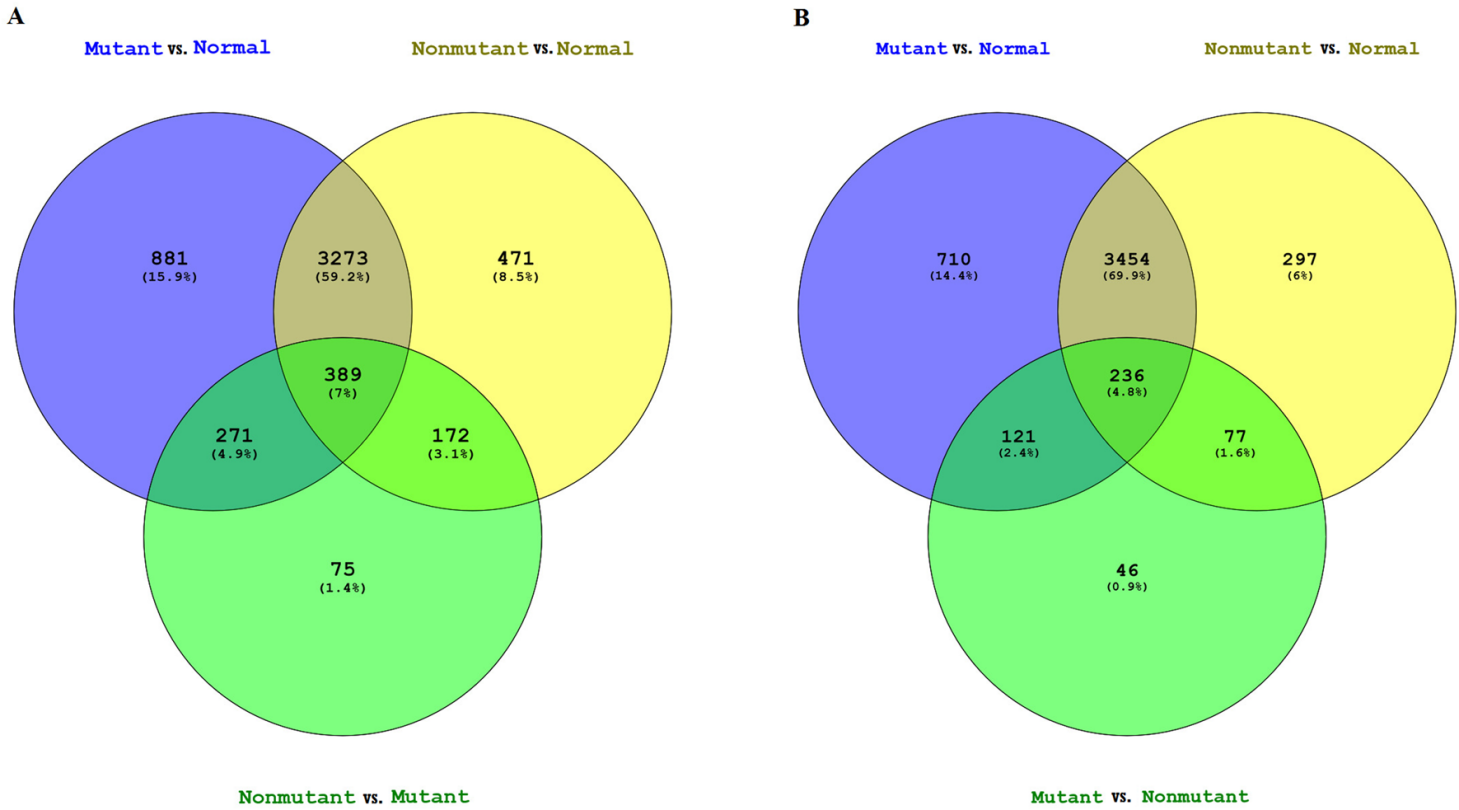
Venn diagram indicating differentially expressed genes overlapping between the samples in (**A**) the entire patient and (**B**) ER-positive subgroup. Blue: *GATA3*-Mutant versus Normal; Yellow: *GATA3*-Non-mutant versus Normal; Green: *GATA3*-Mutant versus Non-mutant.

Venn diagram shows the common and specific genes in every group. As it can be seen in Fig. 2, 389 and 236 genes are common in the three groups of all and ER-positive patients, respectively, that might be involved in breast carcinogenesis and also be influenced by *GATA3* mutations.

### Functional annotation analysis of differentially expressed genes

To gain an insight into the functionality of the DEGs between normal and tumor (mutant and non-mutant) samples, gene set enrichment analysis was performed using the Metascape and DAVID functional enrichment tool. According to DAVID outputs, 36 pathways found to be significantly different between *GATA3*-mutant and normal samples, 7 pathways had been previously reported as the most important pathways related to breast cancer (*p* ≤ 0.05)^20–22^. Evaluation of non-mutant tumors against normal tissue samples indicated 37 significantly different. Also, 3 different pathways (protein digestion and absorption; Wnt signalling; and cell adhesion molecules) were significantly different between mutant and non-mutant tumor tissues. Analysis of ER-positive patients indicated 37 and 36 significantly different pathways in normal samples in comparison with mutant and non-mutant tumors, respectively. Furthermore, pancreatic secretion pathway was different between mutant and non-mutant tumors. These results are shown in the supplementary information file, Table S3.

### PPI network of module analysis

**Figure 3:**
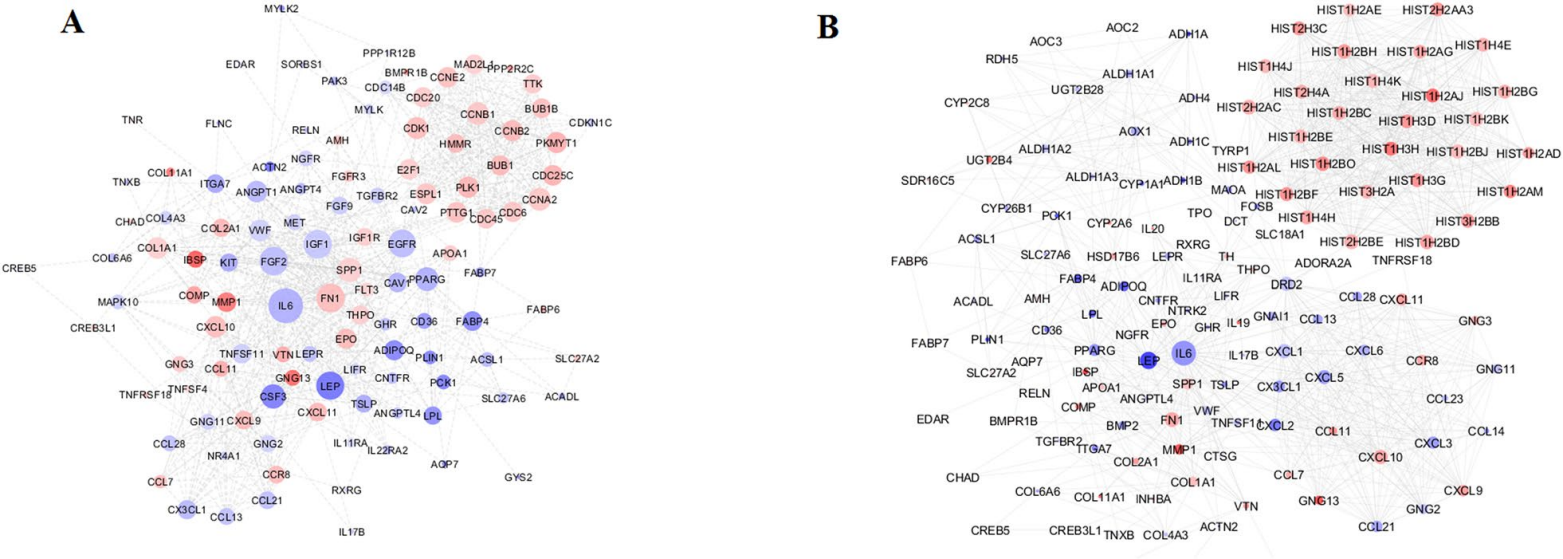
PPI network of breast cancer differentially expressed genes (DEGs) between normal and *GATA3* mutant samples in (**A**) the entire patient and (**B**) ER-positive subgroup. The node size is proportional to the degree value as the bigger size means the larger degree value. The color of the node is related to the expression of genes: up regulated genes are shown in Red and down regulated genes are shown in Blue.

**Figure 4:**
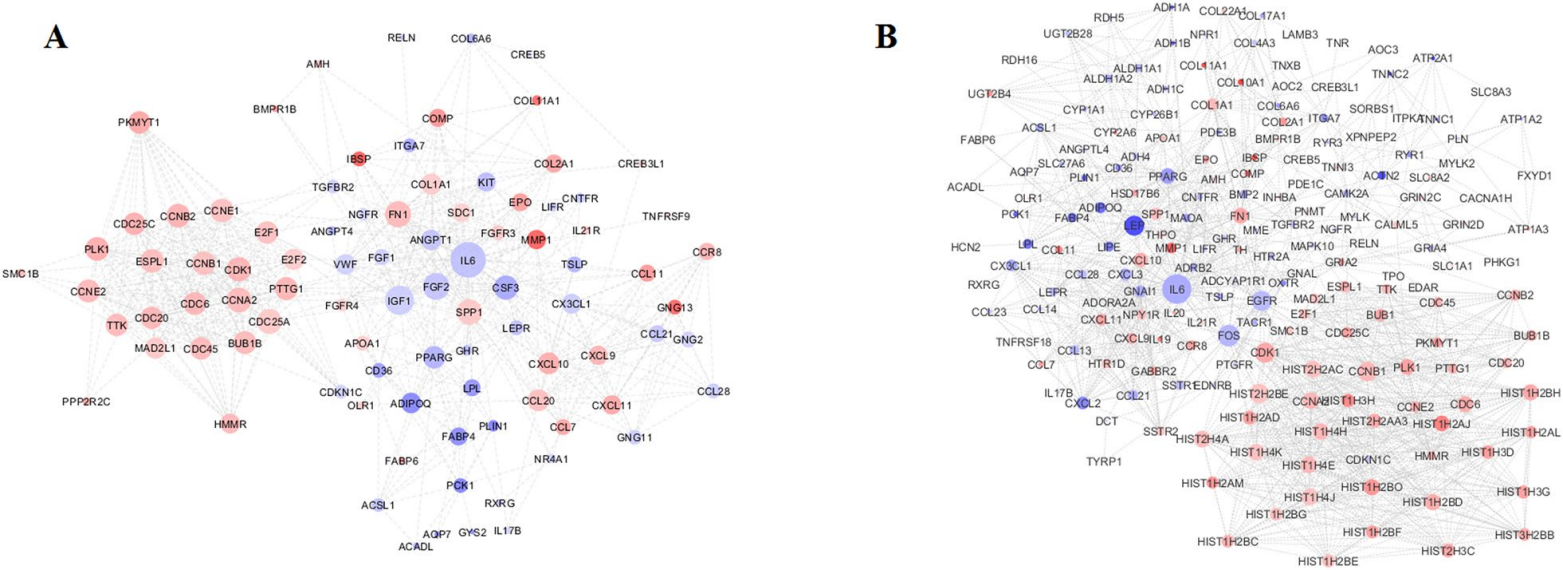
PPI network of breast cancer differentially expressed genes DEGs between normal and *GATA3*-non-mutant in (**A**) the entire patient and (**B**) ER-positive subgroup. The node size is proportional to the degree value as the bigger size means the larger degree value. The color of the node is related to the expression of genes: up regulated genes are shown in Red and down regulated genes are shown in Blue.

To gain a better understanding of the biological relationships between breast cancer-related genes, the genes that share the same GO term related to breast cancer were examined in the STRING database. Results indicated that 116 and 95 genes (proteins) for all patients and 142 and 191 for ER-positive subgroup matched the database and were used to construct the PPI network between *GATA3* mutant tumor and normal tissues (Fig. 3) and between *GATA3* non-mutant tumor and normal tissues, respectively (Fig. 4).

The top nodes with high topology score that were calculated by three centrality methods, were considered as hub nodes. Interleukin 6 (*IL6*) had the highest scores in three centrality methods in both comparisons between normal and mutant and normal and non-mutant groups in all patients as well as ER-positive subgroup. *FN1*, *IGF1*,* FGF2* and *LEP* in all patients and, *LEP* and *FN1* genes in the ER-positive subgroup could be considered as hub nodes in normal and mutant. Moreover, *IGF1*, *FGF2*, *FN1* and *SPP1* genes in all patients and *FOS*, *FGFR*, *LEP* and *CDK1* genes in ER-positive subgroup could be considered as hub nodes in normal and non-mutant groups. PPI analysis did not find any prominent network when the two mutant and non-mutant groups were compared, which may be due to the limited number of identified gene sets.

## Discussion

Cancer, as a multifactorial disease with complex pathological features, is influenced by genetic factors. However, somatic mutations are amongst the most important well-known genetic factors involved in cancer. The role of somatic mutations in tumor development and progression of cancer has been confirmed through advances in technology and increasing knowledge about mutation characteristics. In this study, we focused on the analysis of a gene with known roles in breast cancer, *GATA3*^8,16^, using the large-scale data obtained by the TCGA project^18^. In this cohort, the frequency of somatic mutations in *GATA3* was 14.15%. As previously reported, this gene is one of the three genes representing more than 10% somatic mutations in all breast cancer patients^23^. The analysis of clinical factors in relationship with the *GATA3* somatic mutations reported in TCGA-BRCA project revealed that *GATA3* mutations were associated with several clinical features and pathological subtypes of breast cancer. Also, differential gene expression analysis has identified different patterns of expression in normal samples, *GATA3* mutant and non-mutant tumor tissues in the entire cohort as well as in the ER-positive cases. Furthermore, our results also showed three pathways were significantly different between *GATA3* mutant and non-mutant tumors.

Our results suggested that patients with *GATA3* mutant tumors were significantly younger than those patients without *GATA3* mutations. A previous report has indicated that younger luminal B cases had *GATA3* mutations more frequently than older patients^24^. This finding has also been validated in metastatic breast cancer patients^27^. Since ER-positive younger patients indicated poorer prognosis^28^, a higher rate of *GATA3* mutations may have clinical importance.

Our results suggested the importance of *GATA3* in tumor size in the TCGA dataset. It has been previously reported that mutational load is correlated with the size of tumor in breast cancer patients^29^. Therefore, it is expected to observe a higher rate of *GATA3* mutation in larger tumors. Furthermore, a higher rate of rare types of tumor (Mixed Histology, Mucinous Carcinoma and Medullary Carcinoma) was observed in association with *GATA3* mutations (Table 1). Conversely, after adjustment and also in the ER-positive group, we found a significant difference in mutation status between ILC and IDC, but not in rare types of breast cancer (Table 2). These results may be affected by the small number of rare types in comparison with ductal carcinoma of the breast. However, this may highlight the impact of mutations on different features of breast tumors (Table 2). In addition, the results of our analysis showed ER-positive tumors harbored almost all *GATA3* somatic mutations detected in the patient cohort. This finding confirms previous reports showing an association of *GATA3* with ER-positive status and luminal differentiation, which may reflect its role in response to chemotherapy^30^. Also, a study has shown that *GATA3* up-regulates and stabilizes ER mRNA transcription^31^. In contrast, *GATA3* expression is down-regulated by progestin-induced PR activation^32^. It may explain the association of *GATA3* mutations with the luminal type of breast cancer as a hormone receptor-positive type.

As the two aspects of *GATA3* have been studied, i.e. a difference in expression between mutant and non-mutant or normal tissues and the impact of its mutations on tumor properties, it can be postulated that in agreement with previous studies, our data support the higher level of expression in tumor tissues than normal samples^16,33^ and the lack of importance of *GATA3* somatic mutations as an independent factor in patient survival^11,34^. However, non-mutant samples showed better survival than others in ER-positive patients. METABRIC data indicated the prognostic value of *GATA3 X308_Splice* mutation, as the mutant samples had better survival than wild-type ones both in all patients and ER-positive patients^35^. On the other hand, in samples representing a high expression of *GATA3*, mutant patients had longer survival than wild-types, and mutations in the second *GATA3* zinc-finger (ZnFn2) was associated with lower survival time than other mutations^36^. Another study has also reported that a significant association of *GATA3* mutations with hormone receptor-positive situation may reflect the better prognosis of the disease^17^. All of these different findings suggest the importance of mutation type and co-consideration of other related factors in the association of *GATA3* somatic mutation with overall survival. However, different factors including the number of mutant samples and the study settings may cause this variation. We acknowledge such variation in these findings can make it more difficult to come to a straightforward conclusion. Regarding to the higher level of expression in tumor samples (*GATA3* mutant and non-mutant) than normal ones, a meta-analysis study confirmed the relation between *GATA3* overexpression and favorable phenotypes including ER-positive status^14^. On the other hand, a cell line study indicated the active GATA3 transcription factors cause proliferative phenotypes and promote the growth of ER-positive breast cancer cell lines^37^. In addition to the impact of the mutation on expression level, somatic mutations may affect the binding site and influence the rate of downstream genes expression and result in a changed transcriptional network^36,38^. Furthermore, it has been observed that higher rate of *GATA3* mutations in ER-positive patients may lead to resistance to endocrine therapy^27^. Therefore, all of these findings indicate diverse activities of GATA3 protein which affect the luminal breast epithelial cells via different pathways can neutralize the impact of this gene on the prognosis of the disease.

*MYH2,* as a down-regulated gene in *GATA3*-mutant and non-mutant samples, encodes an Actin-based motor protein with the skeletal muscle contraction activity. According to the Human Protein Atlas^18^, MYH2 protein was not detected in breast tissues, however, low amount of RNA has been observed^39^. Since *GATA3* mutants compared with non-mutant tumor samples did not indicate any difference in expression of *MYH2*, its lower expression in tumor samples may be resulted due to the tumor environment. Similar to *MYH2*, *CKM* (Muscle type of CK) is down-regulated in tumor (*GATA3* mutant and non-mutant) tissues. Expression of this gene in mRNA level has previously been shown in breast samples^39^. Furthermore, a decreased level of serum CK has been specified in breast cancer patients^40^. Moreover, *SMR3B* gene (submaxillary gland androgen-regulated protein 3B) was identified to have differential expression between *GATA3* mutant and non-mutant tumor tissues as mutant samples indicate a lower level of expression. Previously, it has been predicted *SMR3B* has GATA3 transcription factor binding site motif^41^ and is expressed more in triple-negative breast cancer patients with poor prognosis compared to the low-risk patients^42^. As GATA3 protein has a role in expression regulation, lower level of SMR3B expression in tumor carrying *GATA3* mutations can be explained by this fact. *CSN1S1* (Casein Alpha S1), is another top down-regulated gene in *GATA3* mutant samples compared to non-mutants in the ER-positive subgroup. Its RNA expression has been identified in breast tissue, however, the protein has only been detected in lactating breast. Because of significantly different protein expression in benign prostate hyperplasia compared with normal and tumor prostate tissues, *CSN1S1* has been reported as a potential biomarker for early identification of benign prostate hyperplasia patients^43^. Moreover, *CSN1S1* has identified as a tumor suppressor that controls breast tumor growth and metastasis^44^. According to our finding, *GATA3*-mutants had larger tumor size that it may be due to the down-regulation of *CSN1S1*.

We found *MUC2* over-expression in *GATA3*-mutant tumor than normal samples. *MUC2* is up-regulated in mucinous carcinomas^45^, and have higher expression in invasive breast tumors than adjacent normal tissues. A significantly higher level of serum *MUC2* has also been found in breast cancer patients compared with healthy people^46^. Furthermore, as a prognostic effector, MUC2 protein is associated with shorter disease-free survival^47^. Evaluation of a cell line with the limited expression of *MUC2* indicated a decreased rate of proliferation and better response to chemotherapy by efficiently induced apoptosis^48^. These findings confirmed the potential prominent role of *MUC2* expression as the prognostic marker in breast cancer. However, the relationship between *GATA3* and *MUC2* remains to be evaluated. Another up-regulated gene, *S100A15*, is a calcium-binding protein with higher expression in non-mutant tumors than normal ones. While there is evidence which indicates elevated S100A15 transcripts in ER/PR negative breast cancers^49^, the association of this gene with breast cancer prognosis has not been confirmed^50^. In the ER-positive subgroup, *CST5* (Cystatin D), was the first top differentially up-regulated gene between non-mutants and normal. This gene has been down-regulated in colon cancer^51^, and its induction by calcitriol can also prevent the breast cancer cells growth^52^. The mutant and non-mutant comparison showed Aldolase B (*ALDOB*), a glycolytic enzyme, to be up-regulated in *GATA3* mutant samples. However, tumor samples did not show differential expression in comparison with normal ones. Previous studies indicated a decreased level of ALDOB in several cancers^53,54^. Therefore, the higher expression of *ALDOB* in *GATA3* mutant breast cancer tumors may be caused by involved common regulatory pathways that need to be confirmed by functional and gene–gene interaction analyses. Furthermore, according to the Venn diagram, 75 genes in the entire patient group and 46 in ER-positive subgroup, were differentially expressed between *GATA3*-mutant and non-mutant tumors that may indicate the impact of *GATA3* in the expression profile of the tumor cells.

Considering the differently expressed pathways, previously indicated to be associated with breast cancer, protein digestion and absorption pathway was different between all categories^55^. Other pathways were specifically different between mutant and non-mutant tumors. Wnt/β-catenin signaling pathway is a modulating factor of mammary gland morphogenesis and cell properties^20^ and mediates the increase of *GATA3* expression^21^. Consistent with our finding, a previous study indicated WNT/β-catenin signaling as an enriched gene set in *GATA3* X308_Splice mutant breast tumor^35^. Cell adhesion molecules (CAMs) was another different gene set between tumor samples. The role of this pathway has been recognized in the carcinogenesis and metastasis of breast cancer. Therefore, evaluation of the involved genes can be diagnostic, prognostic and therapeutic targets^22,56^. Besides, the regulatory role of *GATA3* in adhesion molecules expression has been identified in cell culture analysis^57^. Hence, expression variation of these genes can happen in association with *GATA3* situation induced by mutations. These findings may reflect the interactions between *GATA3* and genes involved in WNT and cell adhesion molecules pathways in the pathogenesis of breast cancer. Furthermore, we found that systemic lupus (SLE) erythematosus pathway is differentially expressed between ER-positive breast tumor and normal tissues. It has been shown that SLE is influenced by estrogen-estrogen receptor-mediated signaling through the modulation of cytokine production^58^. There are also reports indicating a lower rate of hormone-dependent cancers in SLE patients although they may tend for a higher incidence of triple-negative breast cancer compared to general population^59^. As the main finding of the protein–protein interaction analysis, *IL6* was identified to be an important hub node in the comparison between tumor and normal samples. In line with our results indicating the contribution of this gene in different pathways, IL6 overexpression has been previously described in breast cancer^60^. Many cellular functions including oncogenesis are influenced by IL6^61^. These findings suggest the crucial role of IL6 in the pathogenesis of breast cancer and the importance of targeting this gene in the treatment of the disease.

## Conclusion

In conclusion, our results suggest that *GATA3* mutation status is associated with a number of clinicopathological features, as well as with overall survival time only in ER-positive breast cancer. Our results also indicate a possible common biological process involving *GATA3* mutations and ER/PR status, which needs to be confirmed by functional analyses. The *GATA3* mutations may influence the expression profile of the tumor cells via impact on expression and activity rate of the *GATA3* gene. These findings should also be confirmed using gene–gene interaction analyses and homogenous samples.

## Methods

### Patients and data files

The study population has consisted of female breast cancer patients in the TCGA-BRCA cohort. Information on the *GATA3* mutations in the tumors was retrieved from https://portal.gdc.cancer.gov. This information was available for 975 of the patients. The tumor mRNA expression data (level 3 data; including raw count data) was extracted from Illuminahiseq_rnaseqV2-exon_quantification (MD5) data file at https://gdac.broadinstitute.org/. This data was available for 771 tumor (671 non-mutant and 100 mutant) and 99 normal tissues of the patients. Demographic and clinical data were obtained from the file rendered by the Legacy Archive of the GDC portal at https://portal.gdc.cancer.gov/legacy-archive/files/735bc5ff-86d1-421a-8693-6e6f92055563. Categorization of the study population was performed according to standard protocols^62–68^. All analyses were also replicated in ER-positive samples including 482 non-mutant and 92 mutant tumor tissues.

### Computational analysis of expression profile

The edgeR program (http://bioconductor.org/packages/release/bioc/html/edgeR.html) is a Bioconductor software package for examining the differential expression of replicated count data using an over-dispersed Poisson model and Empirical Bayes methods to account for both biological and technical variability and moderate the degree of over-dispersion across transcripts^69^. This program was used to determine the DEGs in the normal tissues when compared to the tumors (*GATA3* mutant and non-mutant). The probabilistic methods were used by edgeR to evaluate the differential expression. The affected genes determined based on a false discovery rate (FDR) < 0.05 and a log Fold change (FC) > 1.

### Functional annotation of differentially expressed genes (DEGs)

The proteins encoded by DEGs were analyzed, and annotated using Metascape, “A Gene Annotation and Analysis Resource”, which can be used to analyze multi-platform OMICs data (http://metascape.org/gp/index.html), DAVID “Database for Annotation, Visualization and Integrated Discovery” (https://david.ncifcrf.gov/)^70–72^ to test for gene set enrichment analysis, Gene Ontology (GO) terms and pathways. According to the database, DAVID pathways output is based on KEGG (Kyoto Encyclopedia of Genes and Genomes). Only terms with modified Fisher Exact *p* value ≤ 0.05 were considered significant. Metascape is a web-based portal, and is useful for functional annotations of genes^73^.

### Protein–protein interaction (PPI) network

DEGs (corrected *p* values ≤ 0.05) were imported to the search tool of STRING (v10.0, http://string-db.org/) for the retrieval of interacting genes/proteins by selecting Homo sapiens as the organism. STRING can identify a network of close interactions among this set of genes based on information on experimental as well as predicted protein interactions. The three methods including degree centrality, betweenness centrality, and closeness centrality were used to calculate the topology scores of nodes in the PPI network using the CytoNCA^74^.

### Statistical analysis

Demographic and clinical/molecular data that were examined during statistical analyses are shown in the supplementary information file, Table S1. Comparison between variables between the two groups (mutant vs. non-mutant) was examined using Pearson's Chi-squared test for categorical variables and independent sample *t* test for continuous variables. Univariate logistic regression analysis was used to examine the associations of *GATA3* somatic mutation status with different variables, and the odds ratios (OR) and 95% confidence intervals (CIs) were presented. Multivariate logistic regression analysis was used to assess the variables that were independently predictive of the *GATA3* mutation status. For this purpose, covariates with *p* values ≤ 0.05 in the univariate analysis were entered into a multivariable model, excluding the rare variables (ER status and hormone receptor status). In addition, menopause status and age at diagnosis were highly associated, thus, menopausal status (which had more missing data than the age at diagnosis) was excluded from the multivariable model.

Overall survival (OS) time is defined as the time from diagnosis till the time of death or last contact. Associations between variables and OS were examined using the Kaplan–Meier plots/Log-rank test and Cox proportional hazards regression methods. Results of the univariate Cox regression analysis was used to select the variables to be entered into the multivariable Cox regression models. For this purpose, covariates with *p* values less than 0.05 in the univariate analysis were entered into a covariate selection method (Backward-LR), excluding the rare variables, such as metastasis status (pM) and history of neoadjuvant therapy, and highly correlated variables. Highly correlated variables included menopausal status (excluded) and age at diagnosis, tumor size (pT) (excluded) and stage, and lymph node ratio (excluded) and lymph node status (pN). As a result, age, stage, and radiation therapy status were selected for the analysis of the entire cohort. Association of the *GATA3* mutation status with OS was then examined in a multivariable Cox model after adjusting for these clinical factors. Similar to this process, OS analysis was done for the ER-positive subgroup. After excluding the rare variables, such as metastasis status and history of neoadjuvant therapy, and highly correlated variables including menopausal status, tumor size and lymph node ratio, the variables including age, stage, and lymph node status were selected for the assessment of the *GATA3* mutations’ association with OS in a multivariable Cox model. The hazard rate ratio (HR) and 95% CIs were calculated by the Cox models.

A *p* value < 0.05 was considered significant. All statistical analyses were performed using SPSS 16.0 (IBM, USA).

### Ethical approval

This article does not contain any studies with human participants performed by any of the authors.

## Supplementary Information

Supplementary Information.

## Data Availability

The Data belongs to TCGA Research Network and is available in https://www.cancer.gov/tcga.
