# Supplementary material for: GATA3 somatic mutations are associated with clinicopathological features and expression profile in TCGA breast cancer patients
Source: Sci Rep. 2021 Jan 18;11:1679. doi: 10.1038/s41598-020-80680-9 (PMC7814117; doi:10.1038/s41598-020-80680-9)
Supplement: Supplementary file 1 — Supplementary Information. [file 41598_2020_80680_MOESM1_ESM.pdf]

GATA3 somatic mutations are associated with clinicopathological features and expression profile in TCGA breast cancer patients

Running title: _GATA3_ and breast cancer

Fahimeh Afzaljavan [1*], Ayeh Sadat Sadr [2*], Sevtap Savas [3, 4*], Alireza Pasdar [1, 5, 6**]

[1] Department of Medical Genetics and Molecular Medicine, Faculty of Medicine, Mashhad University of Medical Sciences, Mashhad, Iran.

[2] Aquaculture Research Center- South of Iran, Iranian Fisheries Science Research Institute, Agricultural Research Education and Extension Organization (AREEO), Ahvaz, Iran.

[3] Discipline of Genetics, Faculty of Medicine, Memorial University, St. John's, NL, Canada.

[4] Discipline of Oncology, Faculty of Medicine, Memorial University, St. John's, NL, Canada.

[5] Division of Applied Medicine, Medical School, University of Aberdeen, Foresterhill, Aberdeen, AB25 2ZD, UK.

[6] Bioinformatics Research Group, Mashhad University of Medical Sciences, Mashhad, Iran

* Equal contribution

** Corresponding author: Alireza Pasdar

Department of Medical Genetics and Molecular Medicine, Faculty of Medicine, Mashhad University of Medical Sciences, Mashhad, Iran.

E-mail: pasdara@mums.ac.ir , a.pasdar@abdn.ac.uk

Contact phone number: 00985138002310

Fax number: 00985138002287

Table S1. Baseline patient-related data for TCGA samples.

Variable	Categories	All patients	ER-Positive patients
		Frequency (%)	Frequency (%)
**Age**	Mean	58.40±13.22 (26-90)	59.30±13.33 (26-90)
	Age ≤ 35	35 (3.2)	23 (2.9)
	Age > 35	1049 (96.7)	772 (97.0)
	Missing	1 (0.1)	1 (0.1)
	Age ≤ 40	98 (9)	64 (8)
	Age > 40	986 (90.9)	731 (91.8)
	Missing	1 (0.1)	1 (0.1)
**Menopause status[1]**	Peri & Pre	268 (24.7)	109 (23.9)
	Post	705 (65)	529 (66.5)
	Missing	112 (10.3)	77 (9.7)
**Race[2]**	White	749 (69)	568 (71.4)
	Black/African-American	180 (16.6)	107 (13.4)
	Others	62 (5.7)	38 (4.8)
	Missing	94 (8.7)	83 (10.4)
**History of other malignancy**	No	1017 (93.7)	738 (92.7)
	Yes	67 (6.2)	57 (7.2)
	Missing	1 (0.1)	1 (0.1)
**History of neoadjuvant treatment**	No	1070 (98.6)	782 (98.2)
	Yes	13 (1.2)	13 (1.6)
	Missing	2 (0.2)	1 (0.1)
**Margin status[3]**	Negative	907 (83.6)	661 (83.0)
	Positive/Close	110 (10.1)	88 (11.1)
	Missing	68 (6.3)	47 (5.9)
**Lymph node ratio category[4]**	Negative=0	451 (41.6)	303 (38.1)
	Low (>0-0.2)	233 (21.5)	185 (23.2)
	Intermediate (>0.2-0.65)	151 (13.9)	118 (14.8)
	High (>0.65)	82 (7.6)	61 (7.7)
	Missing	168 (15.5)	129 (16.2)
**Tumor size (AJCC pT)**	T1 & T2	905 (83.4)	657 (82.5)
	T3 & T4	177 (16.3)	137 (17.2)
	Missing	3 (0.3)	2 (0.3)
**Lymph node status (AJCC pN)**	Negative	512 (47.2)	352 (44.2)
	Positive	552 (50.9)	428 (53.8)
	Missing	21 (1.9)	16 (2.0)
**Metastasis status (AJCC pM)**	Negative	906 (83.5)	666 (83.7)
	Positive	20 (1.8)	11 (1.4)
	Missing	159 (14.7)	119 (14.9)
**Stage (AJCC stage)**	Stage 1 & 2	796 (73.4)	576 (72.4)
	Stage 3 & 4	265 (24.4)	203 (25.5)
	Missing	24 (2.2)	17 (2.1)
**ER status**	Negative	238 (21.9)	-
	Positive	796 (73.4)	-
	Missing	51 (4.7)	-
**PR status**	Negative	342 (31.5)	121 (15.2)
	Positive	689 (63.5)	672 (84.4)
	Missing	54 (5)	3 (0.4)
**HER2 status[5]**	Negative	764 (70.4)	589 (74.0)
	Positive	161 (14.8)	121 (15.2)
	Missing	160 (14.7)	86 (10.8)
**Receptor status**	ER and/or PR positive (Luminal A, B,C)	813 (74.9)	-
	HER2 overexpressed	35 (3.2)	-

	TNBC	161 (14.8)	-
	Missing	76 (7)	-
**Anatomic neoplasm subdivision**	Left	564 (52)	407 (51.1)
	Right	521 (48)	389 (48.9)
**Histological type of tumor[6]**	IDC	774 (71.3)	531 (66.7)
	ILC	203 (18.7)	190 (23.9)
	Other	107 (9.9)	75 (9.4)
	Missing	1 (0.1)	0 (0.0)
**Vital status**	Alive	934 (86.1)	697 (87.6)
	Dead	151 (13.9)	99 (12.4)
**Chemotherapy**	No	192 (17.7)	180 (22.6)
	Yes	578 (53.3)	394 (49.5)
	Missing	315 (29.0)	222 (27.9)
**Hormone therapy**	No	256 (23.6)	101 (12.7)
	Yes	514 (47.4)	473 (59.4)
	Missing	315 (29.0)	222 (27.9)
**Radiation therapy**	No	423 (39.0)	310 (38.9)
	Yes	574 (52.9)	425 (53.4)
	Missing	88 (8.1)	61 (7.7)
**GATA3 mutation status**	Wild-type	837 (77.1)	582 (73.1)
	Mutant	138 (12.7)	128 (16.1)
	Missing	110 (10.1)	86 (10.8)

[1] Categorized based on (1-3)

[2] Categories based on the information in (4, 5)

[3] Categorized based on the information in (6)

[4] Categorized based on the information in (7)

[5] According to ISH/IHC results

[6] Based on WHO classification (8). Other category includes rare types of tumors (e.g. metaplastic, medullary tumors).

AJCC: American Joint Committee on Cancer; ER: estrogen receptor; IDC: invasive ductal carcinoma; IHC: immunohistochemistry; ILC: invasive lobular carcinoma; ISH: in situ hybridization; PR: progesterone receptor; TNBC: triple negative breast cancer

Table S2. Results of the univariate Cox regression analysis in TCGA data.

Variable	All patients		ER- Positive patients	
	**P-value**	**HR (95% CI)**	**P-value**	**HR (95% CI)**
GATA3 mutation status (Yes vs. No)	0.73	1.09 (0.66-1.80)	0.398	1.26 (0.74-2.15)
Age at diagnosis (Continuous variable)	**1.85E-07**	1.03 (1.02-1.05)	**3.0857E-8**	1.05 (1.03-1.06)
Age (≤35 vs. >35 years)	0.83	0.91 (0.40-2.07)	0.795	1.14 (0.42-3.12)
Age (≤40 vs. >40 years)	0.74	1.09 (0.65-1.83)	0.527	1.28 (0.59-2.78)
Menopause status (Post vs. Peri & Pre)	**0.0007**	2.35 (1.43-3.87)	**0.0003**	3.92 (1.87-8.18)
Race (White as reference)	0.63		0.835	
Black/African-American vs. White	0.44	1.18 (0.78-1.77)	0.549	0.84 (0.46-1.50)
Asian vs. White	0.62	0.75 (0.24-2.36)	0.960	0.00 (0.00-1.19E199)
History of other malignancies (Positive vs. Negative)	0.25	1.49 (0.76-2.94)	0.209	1.65 (0.76-3.58)
History of neoadjuvant therapy (Positive vs. Negative)	**0.04**	3.29 (1.04-10.38)	**0.016**	4.15 (1.30-13.25)
Margin status (Close & positive vs. Negative)	**0.003**	1.96 (1.25-3.07)	**0.046**	1.74 (1.01-3.00)
Lymph node ratio category (Negative as reference)	**5.05E-08**		**0.00003**	1.10 (0.95-1.28)
Low (>0-0.2) vs. Negative	**0.02**	1.71 (1.08-2.71)	0.191	1.47 (0.82-2.63)
Intermediate (>0.2-0.65) vs. Negative	**0.002**	2.14 (1.32-3.47)	**0.035**	1.82 (1.05-3.27)
High (>0.65) vs. Negative	**2.07E-09**	4.84 (2.89-8.11)	**0.000002**	5.28 (2.67-10.43)
Tumor size (T1 as reference)	**0.00007**		**0.015**	
T2 vs T1	0.23	1.29 (0.85-1.94)	0.506	1.18 (0.72-1.95)
T3 vs.T1	0.08	1.58 (0.94-2.67)	0.459	1.27 (0.68-2.38)
T4 vs. T1	**6.16E-06**	4.17 (2.25-7.75)	**0.002**	3.58 (1.61-7.93)
Tumor size category (T3&T4 vs. T1&T2)	**0.003**	1.74 (1.20-2.50)	0.108	1.45 (0.92-2.28)
Lymph node status (Negative as reference)	**2.43E-06**		**0.014**	
N1 vs. Negative	**0.003**	1.78 (1.20-2.64)	0.081	1.53 (0.95-2.47)
N2 vs. Negative	**0.0001**	2.75 (1.65-4.60)	**0.003**	2.50 (1.37-4.58)
N3 vs. Negative	**2.68E-06**	4.14 (2.29-7.48)	**0.040**	2.38 (1.04-5.43)
Lymph node status category (Positive vs. Negative)	**0.00002**	2.15 (1.50-3.08)	**0.009**	1.79 (1.16-2.76)
Metastasis status (M1 vs. M0)	0.18	1.85 (0.76-4.53)	**0.033**	2.98 (1.09-8.18)
Stage (S1 as reference)	**1.96E-13**		**2.2143E-7**	
S2 vs. S1	**0.09**	1.60 (0.92-2.76)	0.719	1.12 (0.61-2.06)
S3 vs. S1	**0.0001**	3.08 (1.73-5.46)	0.053	1.90 (0.99-3.65)
S4 vs. S1	**8.48E-12**	12.29 (5.98-25.25)	**7.6187E-7**	7.81 (3.46-17.63)
Stage category (S3&S4 vs. S1&S2)	**2.04E-08**	2.63 (1.87-3.68)	**0.0002**	2.22 (1.45-3.38)
ER status (Positive vs. Negative)	0.10	0.74 (0.51-1.06)	-	-
PR status (Positive vs. Negative)	0.09	0.75 (0.53-1.05)	0.465	0.82 (0.48-1.40)
HER2 status (Positive vs. Negative)	0.60	1.14 (0.69-1.89)	0.399	1.29 (0.71-2.32)
Receptor status (Luminal type as reference)	0.10			
HER2 overexpression vs. Luminal	0.70	1.22 (0.45-3.32)	-	-
TNBC vs. Luminal	0.03	1.61 (1.05-2.47)	-	-
Laterality (Left vs. Right)	0.20	0.81 (0.59-1.12)	0.711	0.93 (0.62-1.38)
Histological type of tumor (IDC as reference)	0.33		353	
ILC vs. IDC	0.47	0.85 (0.54-1.33)	0.334	0.77 (0.46-1.30)
Others vs. IDC	0.25	1.31 (0.83-2.09)	0.386	1.29 (0.72-2.31)
Chemotherapy (Yes vs. No)	0.72	0.89 (0.47-1.69)	0.202	0.63 (0.31-1.28)
Hormone therapy (Yes vs. No)	0.55	1.19 (0.67-2.13)	0.452	1.49 (0.53-4.22)
Radiation therapy status (Yes vs. No)	**0.01**	0.62 (0.43-0.90)	0.120	0.70 (0.44-1.10)
Involved lymph node (Continuous variable)	**1.84-07**	1.07 (1.04-1.09)	**0.001**	1.06 (1.02-1.10)
Lymph node ratio (Continuous variable)	**6.91E-09**	4.81 (2-83-81.19)	**0.00002**	4.66 (2.31-9.41)

AJCC: American Joint Committee on Cancer; CI: confidence interval; ER: estrogen receptor; HR: hazards ratio; IDC: invasive ductal carcinoma; IHC: immunohistochemistry; ILC: invasive lobular carcinoma; ISH: in situ hybridization; PR: progesterone receptor; TNBC: triple negative breast cancer

Table S3. Most important and significantly enriched Kyoto Encyclopedia of Genes & Genomes (KEGG) pathways in GATA3-mutant tumor tissues vs normal tissues and non-mutant tumor tissues vs normal tissues according to TCGA data.

Pathway	Pathway-definition	Count	P-value	List of genes
**All patients: *GATA3* mutant tumor tissues vs normal tissue**				
hsa03320	PPAR signaling pathway	19	2.12E-05	*LPL, PPARG, RXRG, AQP7, ACADL, ADIPOQ, MMP1, PCK1, ACSL1, APOA1, CD36, SORBS1, PLIN1, SLC27A6, FABP4, FABP7, SLC27A2, ANGPTL4, FABP6*
hsa04512	ECM-receptor interaction	19	7.60E-04	*IBSP, COL4A3, TNXB, COL2A1, VTN, CHAD, HMMR, VWF, CD36, COL6A6, COMP, TNR, ITGA7, RELN, SV2B, COL1A1, COL11A1, SPP1, FN1*
hsa04974	Protein digestion and absorption	18	0.0023	*SLC8A3, COL4A3, SLC8A2, ATP1A3, PRSS1, MME, COL2A1, ATP1A2, XPNPEP2, COL17A1, COL6A6, ACE2, COL1A1, CPA1, CPB1, SLC1A1, COL11A1, COL10A1*
hsa04060	Cytokine-cytokine receptor interaction	35	0.0034	*CSF3, LEPR, CXCL9, CNTFR, KIT, CX3CL1, CXCL11, CCL28, CCL7, CXCL10, IL17B, CCL21, TNFRSF18, GHR, THPO, EPO, EGFR, IL6, TNFSF4, FLT3, TGFBR2, MET, LIFR, EDAR, IL11RA, CCL11, LEP, TSLP, CCR8, AMH, CCL13, TNFSF11, NGFR, BMPR1B, IL22RA2*
hsa04151	PI3K-Akt signaling pathway	45	0.0145	*IBSP, CSF3, FGFR3, FGF9, GNG13, VTN, TCL1B, COL2A1, GNG11, KIT, CHAD, CCNE2, IGF1R, COL6A6, TNR, COMP, CREB3L1, GYS2, GNG2, ANGPT1, GNG3, PPP2R2C, COL11A1, FGF2, GHR, SPP1, ANGPT4, EPO, FN1, EGFR, COL4A3, IL6, TNXB, SGK2, MET, NR4A1, IGF1, CREB5, PCK1, EIF4E1B, VWF, ITGA7, RELN, COL1A1, NGFR*
hsa04110	Cell cycle	20	0.0175	*E2F1, CDC6, CDK1, CDC14B, PKMYT1, TTK, CDC20, ESPL1, PTTG1, CDC25C, CCNB1, CDKN1C, CCNE2, CDC45, CCNB2, MAD2L1, PLK1, BUB1, BUB1B, CCNA2*
hsa04510	Focal adhesion	29	0.0217	*IBSP, CAV2, CAV1, PPP1R12B, COL2A1, VTN, CHAD, IGF1R, COL6A6, PAK3, COMP, TNR, COL11A1, SPP1, FN1, EGFR, COL4A3, TNXB, MET, IGF1, MYLK2, ACTN2, MAPK10, FLNC, VWF, ITGA7, RELN, COL1A1, MYLK*
**All patients: *GATA3* Non-mutant tumor tissues vs normal tissues**				
hsa03320	PPAR signaling pathway	18	1.18E-05	*LPL, OLR1, PPARG, RXRG, AQP7, ACADL, ADIPOQ, MMP1, PCK1, ACSL1, APOA1, CD36, SORBS1, PLIN1, FABP4, SLC27A6, FABP7, FABP6*
hsa04110	Cell cycle	24	8.99E-05	*E2F1, E2F2, CDC6, CDK1, PKMYT1, TTK, CDC20, ESPL1, PTTG1, CDC25C, CDC25A, CCNB1, CDKN1C, CCNE2, CCNE1, CDC45, MAD2L1, CDKN2A, CCNB2, PLK1, BUB1, BUB1B, CCNA2, SMC1B*
hsa04974	Protein digestion and absorption	16	0.0031	*IBSP, TNXB, COL2A1, HMMR, VWF, SDC1, CD36, COL6A6, COMP, TNR, ITGA7, RELN, COL1A1, COL11A1, SPP1, FN1*
hsa04512	ECM-receptor interaction	16	0.0031	*IBSP, TNXB, COL2A1, HMMR, VWF, SDC1, CD36, COL6A6, COMP, TNR, ITGA7, RELN, COL1A1, COL11A1, SPP1, FN1*
hsa04151	PI3K-Akt signaling pathway	40	0.0139	*IBSP, CSF3, FGFR4, FGFR3, GNG13, COL2A1, GNG11, KIT, CCNE2, CCNE1, COL6A6, TNR, COMP, CREB3L1, GYS2, ANGPT1, GNG2, GNG3, FGF1, PPP2R2C, COL11A1, FGF2, SPP1, GHR, ANGPT4, EPO, FN1, IL6, TNXB, SGK2, IGF1, NR4A1, CREB5, PCK1, EIF4E1B, VWF, ITGA7, RELN, COL1A1, NGFR*
hsa04060	Cytokine-cytokine receptor interaction	28	0.0242	*CSF3, LEPR, IL21R, CXCL9, CNTFR, CX3CL1, KIT, CXCL11, CCL28, CCL7, CXCL10, IL17B, CCL20, CCL21, TNFRSF18, GHR, EPO, IL6, TGFBR2, LIFR, LEP, CCL11, TSLP, CCR8, AMH, TNFRSF9, NGFR, BMPR1B*
**All patients: *GATA3* mutant tumor tissues vs Non-mutant tumor tissues**				
hsa04974	Protein digestion and absorption	5	0.0097	*COL9A1, COL9A3, PRSS3, ACE2, COL11A2*
hsa04310	Wnt signaling pathway	5	0.0424	*FZD9, SFRP1, MMP7, WIF1, WNT6*
hsa04514	Cell adhesion molecules (CAMs)	5	0.0462	*MPZ, CLDN6, NTNG1, CLDN10, CDH4*
**ER- positive patients: *GATA3* mutant tumor tissues vs normal tissue**				
hsa05322	Systemic lupus erythematosus	33	8.35E-08	*HIST2H2AA3, HIST1H2BO, HIST1H2BJ, HIST1H2BK, HIST2H2AC, HIST2H4A, C6, C7, HIST1H2AE, HIST1H2AD, HIST1H3G, HIST1H2AG, HIST1H3H, CTSG, HIST3H2BB, HIST1H3D, HIST1H2AM, HIST1H2AL, HIST1H4K, ACTN2, HIST1H2AJ, HIST2H2BE, HIST3H2A, HIST1H2BF, HIST1H2BE, HIST1H4H, HIST1H2BH, HIST1H2BG, HIST1H4J, HIST2H3C, HIST1H2BD, HIST1H4E, HIST1H2BC*
hsa00350	Tyrosine metabolism	14	3.55E-06	*AOC3, AOC2, ADH1C, MAOA, ADH1B, ADH1A, TYRP1, ALDH1A3, ADH4, TPO, DCT, TH, AOX1, HPD*
hsa03320	PPAR signaling pathway	19	1.03E-05	*ACSL1, MMP1, ADIPOQ, AQP7, APOA1, LPL, SORBS1, FABP4, ACADL, FABP6, FABP7, SLC27A6, PPARG, PLIN1, CD36, ANGPTL4, PCK1, RXRG, SLC27A2*
hsa04060	Cytokine-cytokine receptor interaction	40	1.15E-04	*CCL14, CCL13, CXCL6, CNTFR, CXCL9, CCL11, IL20, EPO, CXCL1, CXCL3, CXCL2, CXCL5, CX3CL1, GHR, CCL7, THPO, LEPR, CCR8, TNFSF11, AMH, NGFR, CCL23, CCL21, TSLP, IL11RA, TNFRSF18, IL19, LIFR, INHBA, TGFBR2, TNFRSF10D, EDAR, CXCL10, BMP2, CXCL11, IL6, LEP, BMPR1B, CCL28, IL17B*
hsa00830	Retinol metabolism	16	2.98E-04	*ADH1C, ADH1B, ADH1A, UGT2B28, HSD17B6, SDR16C5, ADH4, CYP2A6, CYP2C8, CYP26B1, ALDH1A2, ALDH1A1, CYP1A1, UGT2B4, RDH5, AOX1*
hsa04512	ECM-receptor interaction	18	0.001152	*TNXB, VWF, COL11A1, FN1, HMMR, COL1A1, COMP, VTN, COL2A1, RELN, SV2B, IBSP, COL4A3, CHAD, SPP1, ITGA7, COL6A6, CD36*
**ER- positive patients: *GATA3* Non-mutant tumor tissues vs normal tissues**				

hsa00350	Tyrosine metabolism	13	5.21E-06	*PNMT, AOC3, AOC2, ADH1C, MAOA, ADH1B, ADH1A, TYRP1, ADH4, TPO, DCT, TH, HPD*
hsa03320	PPAR signaling pathway	18	6.52E-06	*ACSL1, MMP1, ADIPOQ, AQP7, APOA1, LPL, SORBS1, FABP4, ACADL, FABP6, SLC27A6, OLR1, PPARG, PLIN1, CD36, ANGPTL4, PCK1, RXRG*
hsa05322	Systemic lupus erythematosus	26	1.92E-05	*HIST2H2AA3, HIST1H2BO, HIST2H2AC, HIST2H4A, C6, HIST1H2AD, HIST1H3G, HIST1H3H, HIST3H2BB, HIST1H3D, HIST1H2AM, HIST1H2AL, HIST1H4K, ACTN2, HIST1H2AJ, HIST2H2BE, HIST1H2BF, HIST1H2BE, HIST1H4H, HIST1H2BH, HIST1H2BG, HIST1H4J, HIST2H3C, HIST1H2BD, HIST1H4E, HIST1H2BC*
hsa04024	cAMP signaling pathway	31	1.76E-04	*GRIA2, OXTR, ADCYAP1R1, CALML5, NPR1, PDE3B, CAMK2A, ATP1A3, ATP1A2, ADRB2, GNAI1, LIPE, PLN, CREB3L1, TNNI3, CNGA3, AMH, GRIA4, GABBR2, HTR1D, NPY1R, SSTR1, FOS, GRIN2C, SSTR2, GRIN2D, MAPK10, ADORA2A, FXYD1, HCN2, CREB5*
hsa04060	Cytokine-cytokine receptor interaction	35	3.18E-04	*CCL14, CCL13, CNTFR, CXCL9, CCL11, IL20, EPO, CXCL3, CXCL2, CX3CL1, GHR, CCL7, THPO, LEPR, IL21R, CCR8, AMH, NGFR, CCL23, CCL21, TSLP, TNFRSF18, IL19, LIFR, INHBA, TGFBR2, EDAR, CXCL10, BMP2, CXCL11, IL6, LEP, BMPR1B, CCL28, IL17B*
hsa04020	Calcium signaling pathway	28	3.97E-04	*RYR1, PTGFR, MYLK2, OXTR, PDE1C, CALML5, CAMK2A, ATP2A1, ADRB2, HTR2A, CACNA1H, EGFR, RYR3, SLC8A2, MYLK, PLN, EDNRB, PHKG1, TNNC1, TNNC2, TACR1, GRIN2C, GRIN2D, SLC8A3, P2RX6, GNAL, ADORA2A, ITPKA*
hsa04974	Protein digestion and absorption	17	8.07E-04	*COL17A1, CPA1, CPB1, MME, COL11A1, COL22A1, SLC1A1, ATP1A3, ATP1A2, SLC8A2, SLC8A3, COL1A1, COL2A1, XPNPEP2, COL4A3, COL10A1, COL6A6*
hsa00830	Retinol metabolism	14	8.41E-04	*ADH1C, ADH1B, ADH1A, UGT2B28, HSD17B6, ADH4, CYP2A6, CYP26B1, ALDH1A2, ALDH1A1, RDH16, CYP1A1, UGT2B4, RDH5*
hsa04512	ECM-receptor interaction	16	0.002029	*TNXB, LAMB3, COL11A1, FN1, HMMR, COL1A1, COMP, COL2A1, RELN, IBSP, COL4A3, SPP1, TNR, ITGA7, COL6A6, CD36*
hsa04110	Cell cycle	20	0.002321	*CDKN1C, PLK1, BUB1B, TTK, CDC6, PKMYT1, CDC25C, SMC1B, CCNA2, CDC20, CCNB2, CCNB1, CDC45, ESPL1, CCNE2, PTTG1, E2F1, CDK1, BUB1, MAD2L1*
**ER- positive patients: *GATA3* mutant tumor tissues vs Non-mutant tumor tissues**				
hsa04972	Pancreatic secretion	4	0.009735	*CA2, PLA2G2A, CLCA2, SLC4A4*

Table S4. Results of the univariate and multivariable Cox regression analysis for *GATA3* frameshift mutation status

Cox regression	All patients			ER-Positive patients		
	**Variable**	**P-value**	**HR (95% CI)**	**Variable**	**P-value**	**HR (95% CI)**
**Univariate**	*GATA3* mutation status (Yes vs. No)	0.260	1.39 (0.78-2.48)	*GATA3* mutation status (Yes vs. No)	0.130	1.61 (0.87-2.97)
**Multivariable**	*GATA3* mutation status (Yes vs. No)	0.169	1.62 (0.81-3.22)	*GATA3* mutation status (Yes vs. No)	**0.005**	2.56 (1.32-4.95)
	Age at diagnosis	**0.000124**	1.03 (1.02-1.05)	Age at diagnosis	**2.5344E-8**	1.06 (1.04-1.08)
	Stage category (S3&S4 vs. S1&S2)	**2.0517E-10**	4.37 (2.77-6.89)	Stage category (S3&S4 vs. S1&S2)	**0.017**	2.02 (1.13-3.62)
	Radiation therapy status (Yes vs. No)	**0.003**	0.50 (0.31-0.79)	Lymph node status category (Positive vs. Negative)	0.159	1.56 (0.84-2.88)

CI: confidence interval; HR: hazards ratio.
